# The Impact of Precipitation Regimes on Forest Fires in Yunnan Province, Southwest China

**DOI:** 10.1155/2014/326782

**Published:** 2014-08-27

**Authors:** Feng Chen, Shukui Niu, Xiaojuan Tong, Jinlong Zhao, Yu Sun, Tengfei He

**Affiliations:** College of Forest Science, Beijing Forestry University, Beijing 100083, China

## Abstract

The amount, frequency, and duration of precipitation have important impact on the occurrence and severity of forest fires. To fully understand the effects of precipitation regimes on forest fires, a drought index was developed with number of consecutive dry days (daily precipitation less than 2 mm) and total precipitation, and the relationships of drought and precipitation with fire activities were investigated over two periods (i.e., 1982–1988 and 1989–2008) in five ecoregions of Yunnan Province. The results showed that precipitation regime had a significant relationship with fire activities during the two periods. However, the influence of the drought on fire activities varied by ecoregions, with more impacts in drier ecoregions IV-V and less impacts in the more humid ecoregions I–III. The drought was more closely related to fire activities than precipitation during the two study periods, especially in the drier ecoregions, indicating that the frequency and the duration of precipitation had significant influences on forest fires in the drier areas. Drought appears to offer a better explanation than total precipitation on temporal changes in fire regimes across the five ecoregions in Yunnan. Our findings have significant implications for forecasting the local fire dangers under the future climate change.

## 1. Introduction

Forest fires are serious problems and have caused enormous social, environmental, and economic damages in China [1]. Yunnan in Southwest China has experienced a large number of forest fires over the past decades. During 1951–2000, there were on average 2674 fire incidents per annum, with an annual average of burned areas at 111.85 million hm^2^ and annual average casualty involving the death of dozens of people, incurring an average economic loss of more than 10 million dollars directly related to forest fires annually in Yunnan province [2, 3].

Fire behaviors are affected by many factors, such as weather conditions [4], human activities [5], fuel characteristics [6, 7], fire management activities [8], and changes in land uses [9] and climate [10, 11]. Among these factors, climate change is considered to be a key factor attributing to forest fires [12, 13].

Whereas longer-term (decadal or longer) climatic variations can have a great impact on fuel types and loads, interannual climatic variations are likely to influence fire regimes through their effects on fuel desiccation [14–16]. Previous studies have demonstrated that short-term (seasonal to annual) changes in precipitation regimes, which modulate fuel moisture content, are linked to wildfire variability [17–19]. Pausas [20] analyzed the link between forest fire occurrence and climatic variables in the Valencia region of Spain and concluded that summer rainfall is an important factor determining the amount of area burned in that region. Heyerdahl et al. [21] show that widespread fire in the inland Northwest is synchronous with changes in the frequency of warm-dry versus cool-wet spring-summer climate. Carcaillet and Richard [22] suggest that fire regime is related to changes in the seasonality of precipitation in eastern Canada. Multicentury fire histories in the Southwest America demonstrated a strong correlation between low precipitation and years of widespread fire [17]. Analysis of annual area burned in Portugal from 1975 to 1992 and precipitation amounts registered in Coimbra indicates that there is an exponential relationship between annual area burned and total rainfall from May to September [23]. Years of widespread burning in forests of Pacific Coast states before 20th century fire exclusion are found to be broadly synchronous and usually associated with drought conditions [24–26].

A number of studies have demonstrated the importance of anomalous precipitation periods for fire occurrence [13, 27]. In most forests in the North America, fires concurrently occur during intervals when precipitation is low and after a long sequence of days with less than 1.5 mm of rain [22]. Ignition and spread of fires in the boreal forest mainly occur when high pressure dominates for at least 3 days with less than 1.5 mm of precipitation [28].

Precipitation variability can exert greater influence than total precipitation on some ecological processes [29–31]. A previous study on spatial differences in fire regimes across the central Appalachian Mountains shows that intra-annual precipitation variability influences fire occurrence more strongly than total annual precipitation [32]. A variable precipitation regime with long rain-free intervals would generate more days per year that favors burning than a climate with more consistent precipitation and short rain-free intervals, even with identical annual precipitation [33]. Therefore, it is necessary to understand how precipitation shapes fire regimes with consideration of not only the total amount but also the frequency, distribution of precipitation.

Our main hypotheses are as follows: (1) fire number and area burned are correlated to drought and precipitation in Yunnan province, (2) this correlation differs with respect to ecoregion. The major objective of this study, therefore, is to examine the correlation of total precipitation, the frequency and the duration of precipitation with fire activity, and how the correlation may vary by ecoregion, based on historic fire data and observed precipitation data from local weather stations.

## 2. Materials and Methods

### 2.1. Study Area

Yunnan province is located in the southwestern China (21°9′–29°15′N, 97°30′–106°E). The terrain from the northwest to the southeast presents a steeply downward trend and elevations vary from 1000 to 4000 m. The region is characterized by tropical monsoon climate, with warm and humid summer and dry winter. The longer-term annual mean air temperature, monthly mean air temperature of the coldest month (January) and the warmest month (July) of the year are 15°C, 21°C, and 7°C, respectively. The mean annual rainfall is 1100 mm, and precipitation in winter and spring accounts for only about 20% of the annual total.

There are varieties of vegetation types from north to south, including alpine meadow, montane and subalpine temperate forests, subtropical forests, and tropical rainforests. The dominant tree species include* Pinus yunnanensis*,* Pinus kesiya*,* Pinus armandii*,* Picea asperata*,* Abies faber*,* Tsuga chinensis*,* Cyclobalanopsis glauca*, and* Betula alnoides*. The shrubs have an extensive distribution, which are often interspersed with trees and prone to fires. The grasslands are rare except in the alpine areas of northwestern Yunnan. In addition to dominated broad-leaved forests in the Southwest Yunnan, most areas are mainly dominated by coniferous forests which cover an area of 4.53 million hectares and account for 48.6% of the total forest area in Yunnan province. These coniferous pure forests, along with those timbers planted in recent years, are flammable, making the fire risk very serious during the dry season in the region.

**Figure 1:**
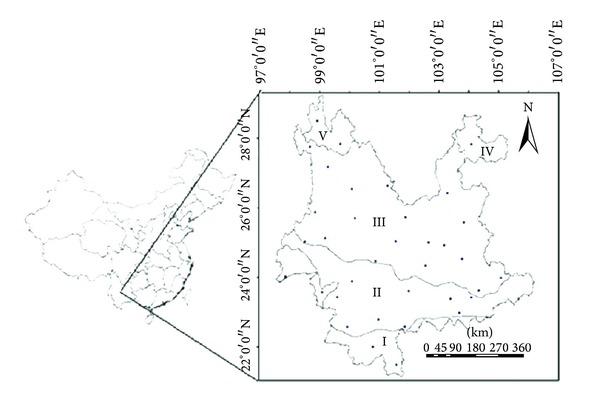
Location of the study area, the dots mark the locations of the observation stations with meteorological data for five ecoregions in Yunnan province.

The study area is divided into five ecoregions, according to the spatial heterogeneity in climate, vegetation types, topography, and so on (Table 1 and Figure 1). Each ecoregion has its distinct climatic characteristics, vegetation structure, and composition. Montane ecoregions have cold and dry climate, while lower-elevation ecoregions are usually characterized by warm and humid climate. Generally, the higher-elevation ecoregions in the north have higher proportions of coniferous forests, while the lower-elevation ecoregions in the south have higher proportions of evergreen broad-leaved forest.

Forest fire in Yunnan province mainly occurs in mid-February to mid-May and fire season is from November to May of the following year. The vast majority of fires are surface fire, accounting for over 95% of total fire incidents. Crown fire, in spite of fewer occurrences, can cause great damage to forests and usually occurs in coniferous forests, especially in young forest stands. In addition, the northwest spruce and fir forests, owning to the larger surface fire, sometimes are affected by crown fire. Therefore, Yunnan province is one of the major fire zones in China.

### 2.2. Climate and Fire Data

The meteorological data used in this study was obtained from 36 official meteorological stations of China Meteorological Data Sharing Network (Figure 1), including daily rainfall data during the period 1982–2008. The prefectures used in this study were chosen according to the spatial distribution of the official meteorological stations and the availability of meteorological data in each station.

During the study period, in total 31,014 forest fires occurred in Yunnan, with burned area of 688,644 ha. The majority of forest fires occurred in winter and spring, and in the spring months fires were more frequent. In this paper, the fire season is defined as the period from December to May of the following year. The daily forest fire data were provided by the Forest Fire Prevention Headquarter Office of Yunnan province, including the timing of fire initiation, fire location, and area burned, and so forth.

### 2.3. Construction of the Drought Index (D)

A dry day is defined as a day with daily precipitation less than 2 mm, which closely fits the estimates of maximum water holding capacity of various forest canopies. Although the threshold value of 2.0 mm for defining a dry day may be fortuitous, it is interesting that this independently determined value occurs at just the point at which precipitation begins to penetrate the canopy and reach the ground.

Weights were assigned to each day in the sequence according to the length of the drought episode. The degree of the drought is related with not only total precipitation but also the frequency and the duration of precipitation, which concerns the distribution of dry days under certain rainfalls, so the drought index during fire season can be expressed as follows:

(1) $$\begin{array}{c} D=\frac{\sum_{i=1}^{n}C_{i}\left(C_{i}+1\right)}{2P}, \end{array}$$

where *D* is the drought index; *C*_*i*_ is the number of consecutive dry days in the *i* sequence, *i* = 1,2,…, *n*, and *n* is the total number of dry sequences and *P* is the total amount of precipitation during fire season.

### 2.4. Data Analysis

The Mann-Kendall test was used to detect a possible fire regime shift and identify a threshold of the change in the fire statistics (number of fires and area burned). The test is used for identifying the threshold in the relationship among variables and attempts to find a point along a distribution of values where the characteristics of the values before and after the point are different [34, 35]. In order to localize the timing of an abrupt fire regime change, a graphical representation of curves *U*(*t*_*i*_) (direct series) and *U*′(*t*_*i*_) (retrograde series) was produced. Direct series are defined as

(2) $$\begin{array}{c} U\left(t_{i}\right)=\frac{t_{i}-E\left(t_{i}\right)}{\sqrt{\mathrm{Var}\left(t_{i}\right)}}, \end{array}$$

where *U*(*t*_*i*_) is the trend value of *t*_*i*_, *t*_*i*_ is the statistical test given by the expression ∑_*i*_ *n*_*i*_, *E*(*t*_*i*_) is the mean of *t*_*i*_ given by the expression *i*(*i* − 1)/4, Var⁡(*t*_*i*_) is the variance of *t*_*i*_ given by the expression *i*(*i* − 1)(2*i* + 5)/72, and *i* is the order of the year in the time series.

The retrograde series are defined as

(3) $$\begin{array}{c} U^{\prime }\left(t_{i}\right)=-U\left(t_{i}\right)\;\text{which gives}\;\;U^{\prime }\left(t_{1}\right)=-U\left(t_{n}\right). \end{array}$$

The intersection of the two curves *U*(*t*_*i*_) and *U*′(*t*_*i*_) localizes the change point. This point is located between the critical values at the 0.05 significance level.

Simple linear regression was applied to show the long-term annual trends in precipitation and *D* values during fire season in each ecoregion for the whole period 1982–2008. Possible differences in the rate of increase or decrease of precipitation and *D* values among the five ecoregions were investigated by testing the regression slopes with analysis of covariance (ANCOVA) [36]. The coefficients of determination (*r* ^2^) were computed from the regression model. Mann-Whitney *U* test at *p* < 0.05 was applied to fire data, to investigate the hypothesis that fire occurrence and area burned significantly decreased after 1988, compared to previous years. *F*-test was used as a criterion for the significance of the trends at significance levels of *p* < 0.05, with 134*df*.

The number of fires and area burned was correlated to precipitation and the *D* values by using a nonparametric correlation test (Spearman's coefficient). The significance levels were adjusted to *p* < 0.0025 by Bonferroni test correction. The statistical analysis was conducted with the SPSS 17.0 statistical software package.

## 3. Results

### 3.1. Variations in Fire Occurrence and Area Burned

**Figure 2:**
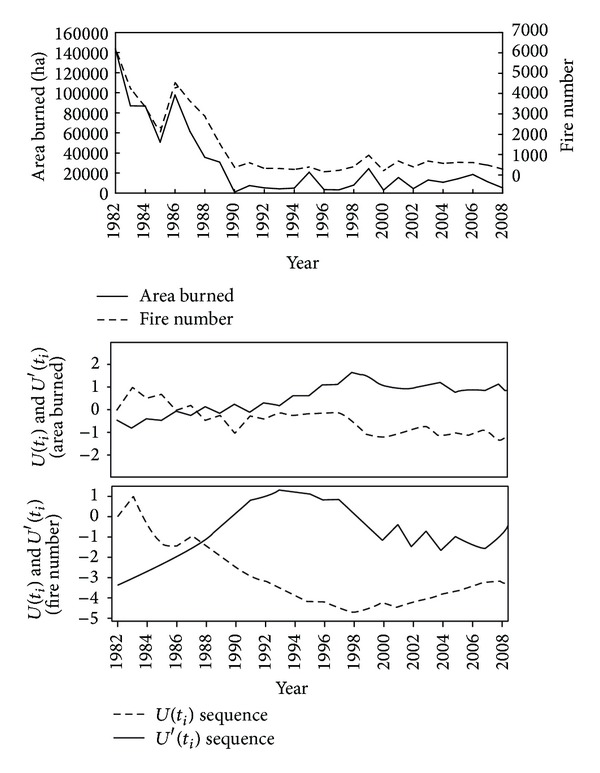
Changes of the annual number of fires and area burned in Yunnan province during the period 1982–2008.

Figure 2 shows the changes of fire statistics in Yunnan during the period 1982–2008. A threshold change was detected for both the number of fires and the area burned in Yunnan beyond the year 1988. The number of fires decreased in Yunnan province during the period 1982–2008. Specifically, fire occurrence decreased in ecoregions I, II, and III. A weaker decrease was observed in ecoregions IV and V.

**Figure 3:**
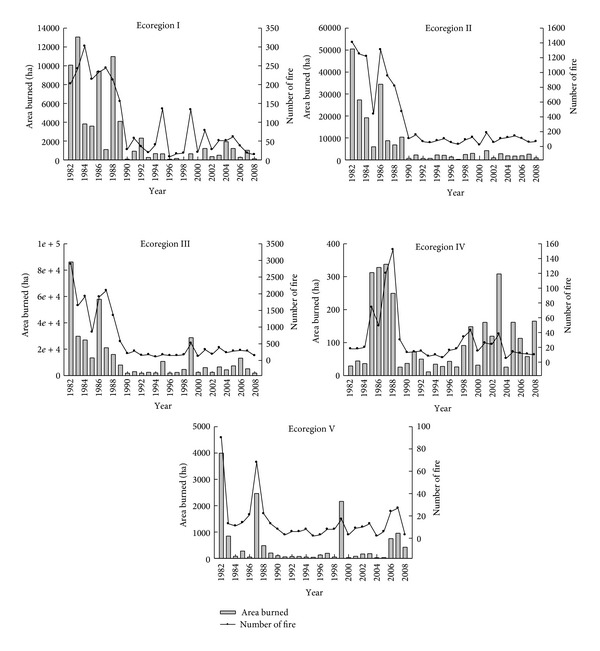
The changing trend of the annual number of fires and area burned for five ecoregions in Yunnan province, during the period 1982–2008.

The number of fires decreased in Yunnan during the period 1990–2008 compared to the period 1982–1988. Particularly, in ecoregions I–III, the mean annual number of fires during the period 1982–1988 was significantly higher than during the period 1990–2008. Similar trends were also observed in ecoregions IV and V (Table 2, Figure 3).

The area burned also followed a decreasing trend all over Yunnan during the period 1982–2008 (Figure 2). The area burned decreased markedly in ecoregions I, II, and III (*p* < 0.01), but not in ecoregions IV and V (Figure 3). Additionally, the mean annual area burned was significantly higher during the period 1982–1988 than during the period 1989–2008 in all ecoregions but IV and V (*p* < 0.001) (Table 2).

### 3.2. Variations in Drought and Precipitation

**Figure 4:**
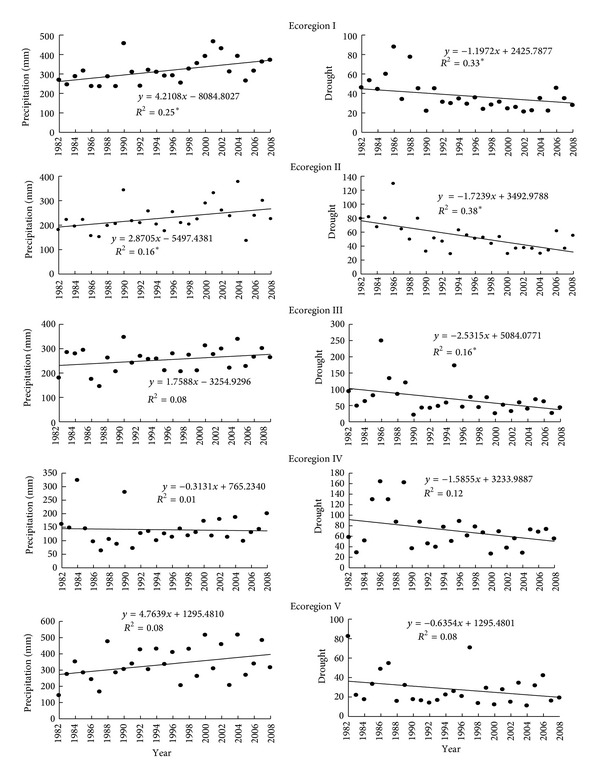
Linear trend of the annual drought episodes and precipitation amounts during fire season for five ecoregions in Yunnan province from 1982 to 2008; the dependent variable is the year and the independent variable is precipitation or drought index value (Formula 2); *denotes significance at the 0.005 level; the significance levels was adjusted by Bonferroni test correction.

The drought index value (*D*) decreased for the whole Yunnan, suggesting that there was a decrease in drought episodes in the fire season during the period 1982–2008. Especially, *D* values decreased significantly in ecoregions I (*p* < 0.005, *R* ^2^ = 0.33), II (*p* < 0.005, *R* ^2^ = 0.38), and III (*p* < 0.005, *R* ^2^ = 0.16) during the study period (Figure 4).

Precipitation showed an increasing trend for the whole Yunnan except for ecoregion IV from 1982 to 2008, with a significant increase in precipitation in ecoregions I (*p* < 0.005, *R* ^2^ = 0.25) and II (*p* < 0.005, *R* ^2^ = 0.16), a weak increase in ecoregions III (*p* = 0.12, *R* ^2^ = 0.08) and V (*p* = 0.11, *R* ^2^ = 0.08), and a slight decrease in ecoregion IV (*p* = 0.36, *R* ^2^ = 0.01) (Figure 4).

The *D* values decreased significantly and the precipitation amounts increased markedly in ecoregions I–III (*p* < 0.005), with no apparent changes in ecoregions IV-V. This indicated that drought episodes decreased significantly in ecoregions I, II, and III (*p* < 0.005), but without showing apparent changes in ecoregions IV and V.

### 3.3. Relationships of Drought and Precipitation with Forest Fire

The correlation between the drought and fire activity (fire number and area burned) was positively significant in all ecoregions but the ecoregion I during the period 1982–1989, and the same was true with precipitation. Both drought and precipitation were significantly related to fire activity in all five ecoregions (*p* < 0.0025) during the period 1989–2008. This suggested that precipitation regime had a significant influence on fire behavior in Yunnan province during the periods.

A comparison of correlations of drought and precipitation with fire activities across ecoregions suggested that drought was better related to fire activities than precipitation, especially in ecoregions IV-V. This indicated that drought can offer a better explanation than total precipitation on the temporal changes in fire regimes in Yunnan province, particularly in drier ecoregions.

## 4. Discussion

The number of fires and area burned showed a significant decreasing trend in Yunnan for the period 1982–2008, with an abrupt change in 1988 (Figure 2). This was mainly attributed to the changes in fire prevention policy implemented in the late 1980s. After the occurrence of the disastrous fire which caused large losses to forest resources and life safety in Daxinganling forest region in 1987, the government strengthened the forest fire prevention program and management for firefighting. It contributed to a decrease in human-induced fire.

A significant increase in precipitation and decrease in drought episodes during the fire season were observed in ecoregions I–III during the period 1982–2008 (Figure 4). These ecoregions are characterized by higher annual precipitation and mean temperature compared to other ecoregions in Yunnan. Ecoregions IV and V did not show any significant change in drought events during the same period. Ma et al. [37] found that precipitation increased significantly in Yunnan during the period 1961–2000, which was not in consistence with global climate change. Peng et al. [38] and Tao et al. [39] found that the drought in the dry season showed a decreasing trend in Yunnan during the period 1961–2005.

There were significant positive correlations among drought, fire occurrence, and area burned in Yunnan province during the period 1982–2008, with exception of ecoregion I for the period 1982–1988 (Tables 3 and 4). Swetnam [27] demonstrated that the regionally synchronous fire occurrence was inversely related to yearly fluctuation in precipitation. Piñol et al. [40] reported significant correlations between the number of very high-risk days and the number of wildfires and area burned in coastal Eastern Spain. A significant correlation between drought and the number of fires and area burned was also reported in the USA [41]. Pausas [20] found a stronger relationship between the area burned and annual (especially summer) rainfall. Westerling et al. [13] showed that the interannual variability of wildfire frequency in the Western United States was strongly associated with regional anomalously dry periods.

The results of correlation analysis suggested that drought had a significantly increasing influence on fire behavior from ecoregion I through V and was much better related to fire activities than precipitation, especially in ecoregion IV-V, during the different periods (Tables 3 and 4). It is likely due to the differences in wet/dry conditions among ecoregions although there were differences in the vegetation and the topography. The level of drought for a period of time was related to not only total precipitation but also the frequency and the duration of precipitation. In particular in the drier regions, total precipitation does not reflect the effect of the consecutive drought which is contingent on the frequency and the duration of dry days. Flannigan and Harrington [42] reported that it was not precipitation but the dry consequence of daily rainfall amount within 1.5 mm that was closely related to the monthly area burned by wildfire from May to August. Lafon and Grissino-Mayer [32] demonstrated that interannual precipitation variability influenced fire occurrence more strongly than total annual precipitation in the central Appalachian Mountains. Lafon and Quiring [33] suggested that a variable precipitation regime should promote fire because it comprised episodic heavy rainfall events separated by multiple rain-free days that permitted fuels to dry and burn.

Drought is a key factor of wildfire occurrence [41, 43]. Drought can desiccate forest fuel and decrease fuel moisture content, making it more ignitable, and, hence, increase the probability of fuel ignition. Many studies have demonstrated strong relationships between the moisture of the dead fine fuel and wildfire occurrence by human activities [44, 45]. Although drought severely exacerbates burning conditions, wind is the primarily environmental factor that influences area burned by wildfires [46–48]. Wildfires can quickly grow to large sizes when hot and dry conditions coincide with wind events [49, 50].

The shifts (decrease) in fire regime in Yunnan may be not ecologically good for some regions especially in which there is a high proportion of forest. The decrease in fire number and area burned can contribute to accumulation of a large amount of forest fuel, which can increase the potential of severe fire in the coming years. Therefore, prescribe burning is needed to prevent major fire occurrence in these regions.

## 5. Conclusions

Conclusively, the precipitation regime had significant impact on the number of fires and area burned in Yunnan province during the period 1982–2008. The constructed drought index offered a better explanation than total precipitation for the temporal changes in fire regime across ecoregions of Yunnan. The frequency and the duration of precipitation should be considered when the relationship between the fires and climate are studied, especially in dry areas. The incorporation of the precipitation regime into a predictive model of forest fire activity could be the focus of future research.

## Figures and Tables

**Table 1:** Climate, vegetation, and topography for five ecoregions in Yunnan province.

Ecoregion	Elevation rage (m)	Mean annual precipitation (mm)	Climatic type	Vegetation type
I	1000–1800	1300–2000	Wet	Tropical seasonal rainforest
II	1500–2000	1000–1600	Semiwet	Subtropical evergreen broadleaved forest
III	1800–3600	800–1000	Semiwet	Subtropical evergreen broadleaf and coniferous forest
IV	3000	800–900	Semidry	Subtropical evergreen broad-leaved forest
V	4000	600–700	Semidry	Boreal coniferous forests,meadows

**Table 2:** Mann-Whitney *U* test for the number of fires and area burned in Yunnan province during the two periods (1982–1988, 1989–2008).

Number of fires	I	II	III	IV	V
Period of 1982–1989	225 (42)	982 (377)	1651 (734)	76 (90)	37 (43)
Period of 1990–2008	45 (37)	91 (44)	228 (103)	18 (11)	9 (7)
Change of mean (%)	−80	−90.7	−86.2	−76.3	−75.7
*U*	0.000	0.000	0.000	19	28
Significance level	*p* < 0.001	*p* < 0.001	*p* < 0.001	*p* = 0.001	*p* = 0.009

Burned area (ha)	I	II	III	IV	V

Period of 1982–1989	9189 (4439)	20395 (15962)	32410 (26562)	176 (143)	2799 (6189)
Period of 1990–2008	1121 (1068)	1709 (1067)	5687 (6428)	85 (78)	290 (521)
Change of mean (%)	−87.8	−91.6	−82.5	−51.7	−89.6
*U*	0.000	0.000	7	61	38
Significance level	*p* < 0.001	*p* < 0.001	*p* < 0.001	*p* = 0.449	*p* = 0.044

**Table 3:** Spearman's correlation coefficient between precipitation amounts, drought index values, and fire activity in fire season for five ecoregions in Yunnan province, during the period 1982–1988.

Ecoregion	*D*/*P*	Number of fires	Area burned
I	Drought	0.563	0.587
	Precipitation	−0.521	−0.534

II	Drought	0.687∗	0.596
	Precipitation	−0.663∗	−0.532

III	Drought	0.692∗	0.677∗
	Precipitation	−0.680∗	−0.661∗

IV	Drought	0.801∗	0.785∗
	Precipitation	−0.695∗	−0.683∗

V	Drought	0.792∗	0.815∗
	Precipitation	−0.689∗	−0.662∗

*D* denotes the drought index values; *P* denotes precipitation amounts in fire season; ∗denotes significance at the 0.0025 level; the significance levels were adjusted by Bonferroni test correction.

**Table 4:** Spearman's correlation coefficient between precipitation amounts, drought index values, and fire activity in fire season for five ecoregions in Yunnan province, during the period 1989–2008.

Ecoregion	*D*/*P*	Number of fires	Area burned
I	Drought	0.536∗	0.551∗
	Precipitation	−0.510∗	−0.527∗

II	Drought	0.561∗	0.576∗
	Precipitation	−0.523∗	−0.525∗

III	Drought	0.601∗	0.589∗
	Precipitation	−0.565∗	−0.572∗

IV	Drought	0.683∗	0.697∗
	Precipitation	−0.597∗	−0.576∗

V	Drought	0.743∗	0.762∗
	Precipitation	−0.592∗	−0.602∗

*D* denotes the drought index values; *P* denotes precipitation amounts in fire season; ∗denotes significance at the 0.0025 level; the significance levels were adjusted by Bonferroni test correction.
